# Helicopter Emergency Medical Services (HEMS) over-triage and the financial implications for major trauma centres in NSW, Australia

**DOI:** 10.1186/1471-227X-13-11

**Published:** 2013-07-01

**Authors:** Colman B Taylor, Kate Curtis, Stephen Jan, Mark Newcombe

**Affiliations:** 1 The George Institute for Global Health, Sydney, NSW, Australia; 2 Sydney Medical School, The University of Sydney, Missenden Rd Camperdown, PO Box M201, 2050 Sydney NSW, Australia; 3 Sydney Nursing School, The University of Sydney, Sydney, NSW, Australia; 4 Wollongong Hospital, Wollongong, NSW, Australia; 5 Greater Sydney Area HEMS, Sydney, NSW, Australia

**Keywords:** Wounds and injury, Trauma systems, Helicopter emergency Medical services, Patient acuity, Cost, Reimbursement

## Abstract

**Background:**

In NSW Australia, a formal trauma system including the use of helicopter emergency medical services (HEMS) has existed for over 20 years. Despite providing many advantages in NSW, HEMS patients are frequently over-triaged; leading to financial implications for major trauma centres that receive HEMS patients. The aim of this study was to investigate the financial implications of HEMS over-triage from the perspective of major trauma centres in NSW.

**Methods:**

The study sample included all trauma patients transported via HEMS to 12 major trauma centres in NSW during the period: 1 July 2008 to 30 June 2009. Clinical data were gathered from individual hospital trauma registries and merged with financial information obtained from casemix units at respective hospitals. HEMS over-triage was estimated based on the local definition of minor to moderate trauma (ISS≤12) and hospital length of stay of less than 24 hrs. The actual treatment costs were determined and compared to state-wide peer group averages to obtain estimates of potential funding discrepancies.

**Results:**

A total of 707 patients transported by HEMS were identified, including 72% pre-hospital (PH; n=507) and 28% inter-hospital (IH; n=200) transports. Over-triage was estimated at 51% for PH patients and 29% for IH patients. Compared to PH patients, IH patients were more costly to treat on average (IH: $42,604; PH: $25,162), however PH patients were more costly overall ($12,329,618 [PH]; $8,265,152 [IH]). When comparing actual treatment costs to peer group averages we found potential funding discrepancies ranging between 4% and 32% across patient groups. Using a sensitivity analysis, the potential funding discrepancy increased with increasing levels of over-triage.

**Conclusions:**

HEMS patients are frequently over-triaged in NSW, leading to funding implications for major trauma centres. In general, HEMS patient treatment costs are higher than the peer group average and the potential funding discrepancy varies by injury severity and the type of transport performed. Although severely injured HEMS patients are more costly to treat, HEMS patients with minor injuries make up the majority of HEMS transports and have larger relative potential funding discrepancies. Future episode funding models need to account for the variability of trauma patients and the proportion of patients transported via HEMS.

## Background

Trauma systems facilitate the transport of patients to receive treatment at designated hospitals and have been shown to reduce patient mortality in Australia [1] and internationally [2]. The New South Wales (NSW) trauma system was introduced in 1991, and has been formally monitored since 2002 [3]. Following the trauma system implementation, NSW trauma centres (referred to as major trauma centres) receive higher volumes of trauma and currently admit more trauma patients than any other state/territory in Australia due to its greater population size [4].

In NSW, major trauma centres are funded using the episode funding model. Episode funding uses Australian Refined Diagnostic Related Groups (AR-DRGs) to describe the patient’s illness or injury. Each admitted patient is allocated an AR-DRG classification after hospital discharge. The state-wide average patient costs for each AR-DRG form the basis of hospital funding [5]. However in cases of trauma, many of the AR-DRGs that are typically assigned are not unique to trauma. Within each AR-DRG there can be a wide range of diagnoses, injuries, complexity and severity [6], which potentially leads to underfunding of acute trauma treatment [6-8].

Helicopter Emergency Medical Services (HEMS) have been integrated into trauma systems to provide timely treatment and transport trauma patients to designated hospitals. The acronym HEMS has become synonymous with specialist retrieval systems that may include helicopter, fixed wing and road ambulance transportation. In this study HEMS applies strictly to helicopter transportation. In NSW nine HEMS perform primary scene responses (pre-hospital) and secondary inter-facility transfers (inter-hospital) as part of the NSW trauma system [9]. Within a formal trauma system a HEMS can provide numerous advantages including access to areas without road infrastructure, timely treatment by a specialist physician and/or paramedic and the ability to rapidly transport patients over large distances. During pre-hospital and inter-hospital transport, HEMS typically bypass smaller hospitals, transporting patients to major trauma centres to comply with the aims of the NSW trauma system [10]. Although this practice conforms to local transport protocols, the resource implications for major trauma centres have not been previously investigated.

Like most pre-hospital ambulance services, HEMS transport a proportion of patients with less severe injuries to major trauma centres, known as over-triage [11]. Although this practice negatively influences HEMS cost-effectiveness [12], it is common in practice because it safeguards against under-triage, an outcome that is likely to be medically, politically and societally unacceptable. Given a degree of HEMS over-triage is likely to remain common practice for the foreseeable future, it is important to document its incidence and to assess the relationship between differing levels of over-triage and cost. Further, taking into account the current episode funding model it is important to examine the financial implications of HEMS over-triage to receiving hospitals such as major trauma centres, which receive the majority of HEMS transports.

Using a state-wide sample of HEMS transports, the aim of this study was to investigate the financial implications of HEMS over-triage from the perspective of major trauma centres in NSW. In doing so we provide a description of HEMS patients, estimates of over-triage and a comparison of the true cost of treating HEMS patients at major trauma centres in NSW in relation to peer group averages to assess potential funding discrepancies.

## Methods

### Inclusion criteria

Patients were included in this study if they were admitted to a NSW major trauma centre via HEMS transport and were captured in the respective trauma databases (see ‘Data capture’) during the 2008/2009 financial year. Patients transported by other transport modes such as ambulance or private vehicle were excluded from the sample (see ‘Variable definition and data analysis’ for further information).

### Setting

The characteristics of the NSW trauma system and HEMS in NSW have been previously described [9,13]. As of the 1st July 2008, the NSW trauma care system incorporated a networked system of 23 designated trauma hospitals, which were classified as either major adult (n=9), major paediatric (n=3), regional (n=2) or rural regional (n=10) according to available resources [13].

### Ethics

Multi-site ethics approval (HE09/266) was gained from the South East Sydney and Illawarra Area Health Service (SESIAHS) Health and Medical Health Research Ethics Committee (HREC). Additionally, approval was gained from each participating sites clinical governance unit using the Site Specific Application (SSA).

### Data capture

All 12 hospitals (3 paediatric; 9 adult) designated major (Level I) trauma centres by NSW Ministry of Health at the time of the study collaborated on this project [13]. A minimum data set, including mode of arrival and injuries sustained, was collected on all trauma patients admitted between 1 July 2008 and 30 June 2009 from existing trauma registries. Each trauma centre has an established registry that is maintained by a data manager and overseen by a trauma nurse coordinator. Trauma patients are identified through trauma calls, review of the Emergency Medical Record System and clinical patient rounds.

### Data synthesis and recoding

Due to variance in site databases, the descriptors or codes within each variable required manual review and recoding. Once the data sets were merged, frequencies were performed on each variable. Using a consensus process amongst the co-investigators, terms for each variable were summarised into definitive labels. To ensure consistency across the dataset, the Abbreviated Injury Scale (AIS) codes were validated and AIS98 codes were mapped to AIS05 equivalents [14].

### Costing methods and linkage

Following amalgamation of the final trauma dataset, medical record numbers and admission dates from the data were provided to the casemix or performance units at each health service or hospital to link costing data. The Performance Management Reporting System [15] was used for all patient costing in NSW. Patient costing, including indirect expenses (overheads, human resources using staffing head count, cleaning expense using floor space) was conducted in accordance with 2008–09 NSW Program and Product Data Collection [16]. The 2008–09 state-wide average costs for each AR-DRG (which forms the basis of funding) were obtained from the NSW Ministry of Health Inter-Government and Funding Strategies Branch [17]. To estimate potential funding discrepancies, the hospital level cost data were compared with other NSW hospitals of similar size and resources (‘peer group’) to determine variance within AR-DRGs.

### Variable definition and data analysis

Patients were included in the analysis based on information recorded under mode of arrival in the trauma database. Classification of transport type (pre-hospital or inter-hospital) was based on information recorded in the inter-hospital transfer variable. For increased accuracy and consistency across datasets, information on length of stay and admission to ICU and OR were sourced from cost data. In cases where ICU costing data were not available, we sourced ICU admission information from clinical data.

Injury severity was classified using the Injury Severity Score (ISS), which is an anatomical scoring system that provides an overall score for patients with multiple injuries (range: 1 to 75, with higher scores associated with higher mortality). The ISS combines the Abbreviated Injury Scale injury scores (AIS; range: 1–6), which are assigned across six body regions. In order to define a HEMS over-triage based on injury severity, we used the local definition of minor to moderate injury (ISS≤12 [13]). As a further measure of over-triage we also examined patients discharged within 24 hrs. Head injury was defined according to an anatomical injury to the head (by selecting AIS codes relating to intra-cranial injury). Polytrauma was defined as the presence of an injury in three or more body regions. Data analysis was undertaken in SAS v9.2 [18].

## Results

A total of 707 patient records transported by HEMS to a major trauma centre in NSW were identified, representing 4% of the total patient cohort (N=17,522). The HEMS patient cohort included 71.7% pre-hospital transports (N=507) and 28.3% of inter-hospital transfers (N=200). Cost data were available in the majority of cases (N=684; 96.7%).

### Patient characteristics

Table 1 shows patient demographics stratified by the type of transport performed. Compared to HEMS pre-hospital (PH) patients, HEMS inter-hospital (IH) patients were older (median age: 32 [PH]; 34 [IH]), more severely injured (median ISS: 12 [PH]; 21 [IH]) had longer hospital stays (median LOS: 6.5 [PH]; 14 [IH]) and a higher mortality rate (overall mortality: 5.1% [PH]; 9.0% [IH]). For pre-hospital transports, the major causes of trauma (>10% prevalence) included falls (N=80; 15.8%), motor bike crashes (N=143; 28.2%) and motor vehicle crashes (N=148; 29.2%). The distribution was similar for inter-hospital transfers (falls: N=55; 27.5%; MBC: N=31; 15.5%; MVC: N=38; 19.0%) with violence also contributing 13% (N=26) of cases. Excluding missing cases, the proportions of patients classified with polytrauma (N=87; 53.0%), head injuries (N=28; 18.0%) and ICU admission (N=127; 63.5%) were higher in inter-hospital patients compared to pre-hospital patients (polytrauma: N=210; 45.1%; head injuries: N=13; 8.1%; ICU admission: N=154; 30.4%). Finally, the proportion of patients with an operating room (OR) related cost were similar between pre-hospital transports (N=304; 63.6% excluding missing) and inter-hospital transports (119; 61.7% excluding missing).

**Table 1:** Demographics and clinical characteristics of patients transported by HEMS to major trauma centres in NSW, stratified by type of transport performed

	**Pre-hospital**	**Inter-hospital**
**Total N**	507	200
**Age (Median, IQR; 95% CI) [N missing; %**^**1**^**]**	32 (19, 49; 9–70) [0; 0%]	34 (22, 54.5; 13–82) [0; 0%]
**ISS (Median, IQR; 95% CI) [N missing; %**^**1**^**]**	12 (5, 20; 1–38) [41; 8.1%]	21 (10, 26; 2–41) [36; 18.0%]
**LOS (Median, IQR; 95% CI) [N missing; %**^**1**^**]**	6.5 (2, 18; 1–67) [5; 1%]	14 (5, 26; 1–75) [1; 0%]
**Male (N; %**^**2 **^**[N missing; %**^**1**^**]**	367 (72.4%) [0; 0%]	157 (78.9%) [1; 0.5%]
**Mechanism of Injury (N %**^**1**^**)**		
Animal	25 (4.9%)	6 (3.0%)
Burns	9 (1.8%)	6 (3.0%)
Drowning or submersion	8 (1.6%)	3 (1.5%)
Fall	80 (15.8%)	55 (27.5%)
Industrial accident or machinery	8 (1.6%)	3 (1.5%)
Motor bike accident	143 (28.2%)	31 (15.5%)
Motor vehicle accident	148 (29.2%)	38 (19%)
Pedal cyclist	10 (2.0%)	7 (3.5%)
Pedestrian	23 (4.5%)	9 (4.5%)
Sport	11 (2.2%)	4 (2.0%)
Violence	20 (3.9%)	26 (13.0%)
Other	18 (3.6%)	12 (6.0%)
Missing	4 (0.8%)	0 (0%)
**ICU (N, %**^**1**^**) [N missing; %**^**1**^**]**	154 (30.4%) [0; 0%]	127 (63.5%) [0; 0%]
**OR (N, %**^**2 **^**[N missing; %**^**1**^**]**	304 (63.6%) [29; 5.7%]	119 (61.3%) [6; 3%]
**Head Injury (N %**^**2**^**) [N missing; %**^**1**^**]**	13 (2.8%) [41; 8.1%]	28 (17.0%) [36; 18%]
**Polytrauma (N %**^**1**^**; %**^**2**^**)**		
1 or 2 body regions	256 (50.5%; 54.9%)	77 (38.5%; 47.0%)
>2 body regions	210 (41.4%; 45.1%)	87 (43.5%; 53.0%)
Missing (N, %^1^)	41 (8.1%)	36 (18.0%)
**Died (N, %**^**1**^**) [N missing; %**^**1**^**]**	26 (5.1%) [0; 0%]	18 (9.0%) [0; 0%]

^1^ Percent of total patients.

^2^ Percent of total patients [minus missing].

### Estimates of over-triage

**Figure 1:**
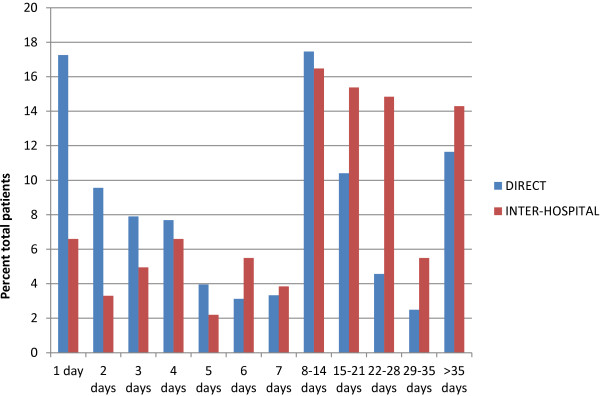
Patient length of stay (excluding deaths) during acute admission to a major trauma centre in NSW.

Based on local NSW criteria (ISS≤12), Table 2 shows 51.1% of pre-hospital patients were transported with minor to moderate injuries, and were therefore considered over-triaged. Inter-hospital transfers had lower rate of minor to moderate injury compared to pre-hospital transports suggesting an over-triage rate of 28.7% (Table 2). Regarding length of stay, Figure 1 shows approximately 17.3% (N=83) of HEMS scene transport patients were discharged within 1 day compared to 6.6% of inter-facility patients (N=12).

**Table 2:** Estimated over-triage rates based on proportion of patients transported with minor to moderate injuries according to local criteria (ISS≤12)

	**Pre-hospital**	**Inter-hospital**
**All patients (N missing ISS; % missing)**	507 (41; 8.1%)	200 (36; 18.0%)
**ISS≤12 (N; %)**^**1**^	238 (51.1%)	47 (28.7%)
**ISS>12 (N; %)**^**1**^	228 (48.9%)	117 (71.3%)

^1^Excluding missing.

### Treatment costs (2009 AUD)

**Figure 2:**
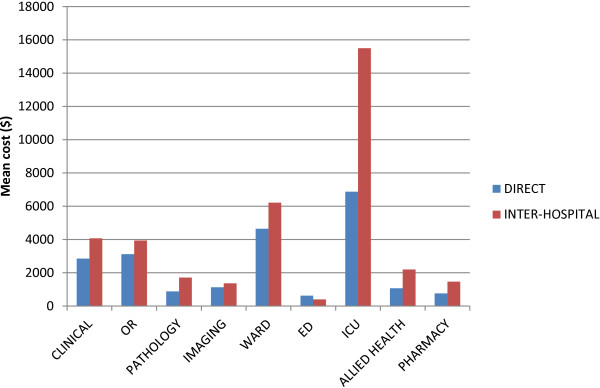
Average cost components contributing to the total cost of acute care at major trauma centres in NSW.

On average, patients transported inter-hospital (IH) were more costly to treat ($42,604) compared to pre-hospital (PH) ($25,162), however given the larger proportion of PH patients, this group were more costly overall ($12,329,618 [PH]; $8,265,152 [IH]) (Table 3). The major contributors to treatment costs were ICU, ward, clinical and OR costs. In particular, ICU costs were the major contributor to the discrepancy between PH and IH patient costs (Figure 2).

**Table 3:** Mean/total actual cost of treatment, peer group average cost and discrepancy between actual cost and peer group average cost, stratified by severity of injury (ISS≤12) and type of transport performed

	**Pre-hospital**		**Inter-hospital**	
	Total	Difference (%)	Total	Difference (%)
**All patients**				
Mean cost (95% CI)	$25,162 ($929 - $102,918)		$42,604 ($1,115 - $166,784)	
Mean peer group average cost (95% CI)	$23,318 ($1,957 - $109,832)	$2,532 (−10.1%)	$40,579 ($2,219 - $109,831)	$2,875 (−6.7%)
Total cost	$12,329,618		$8,265,152	
Total peer group average cost	$11,169,124	$1,197,550 (−9.7%)	$7,750,526	$546,276 (−6.6%)
**ISS ≤12**				
Mean cost (95% CI)	$8,549 ($802 - $27,716)		$18,564 ($850 - $46,592)	
Mean peer group average cost (95% CI)	$7,450 ($1,626 - $19,138)	$1,230 (−14.4%)	$12,683 ($1,539 - $44,490)	$5,881 (−31.7%)
Total cost	$1,966,196		$853,947	
Total peer group average cost	$1,676,169	$271,818 (−13.8%)	$583,435	$270,512 (−31.7%)
**ISS >12**				
Mean cost (95% CI)	$36,622 ($1,284 - $150,941)		$51,676 ($1,115 - $186,238)	
Mean peer group average cost (95% CI)	$36,603 ($3,827 - $109,832)	$1,316 (−3.6%)	$50,595 ($4,460 - $109,832)	$2,803 (−5.4%)
Total cost	$8,056,861		$5,839,397	
Total peer group average cost	$7,833,132	$278,993 (−3.5%)	$5,565,468	$305,579 (−5.2%)

Results were generally consistent when stratified by injury severity. For patients with minor to moderate injuries, average costs were approximately 2-fold lower for PH patients ($8,549) compared to IH patients ($18,564). For patients with severe injuries, average costs were also lower for PH patients ($36,622) compared to IH patients ($51,676). However, after accounting for the proportions of PH an IH transports overall, total costs were lower for IH patients ($853,947 [ISS≤12]; $5,839,397 [ISS>12]) compared to PH patients ($1,966,196 [ISS≤12]; $8,056,861 [ISS>12]) (Table 3).

### Cost variance

Across all patients groups, results showed that the actual costs were consistently higher than the peer group average costs with the discrepancy between the two figures ranging between 4% to 32% overall (Table 3). For pre-hospital (PH) and inter-hospital (IH) transports, the overall discrepancy between actual costs and peer group averages was higher for PH patients compared to IH patients, both in absolute (PH: $1,197,550; IH: $546,276) and relative amounts (PH: 10%; IH: 7%).

When compared by injury severity (according to local criteria), minor to moderate injuries (ISS≤12) had a similar absolute discrepancy overall, between actual total costs and the peer group average total (PH: $271,818; IH: $270,512) compared to severe injures (ISS>12) (PH: $278,993; IH: $305,579). However the relative discrepancies between actual costs and peer group averages were at least 4-fold higher overall, for minor injuries (PH: 14%; IH: 32%) compared to severe injuries (PH: 4%; IH: 5%) (Table 3).

### Sensitivity analysis

**Figure 3:**
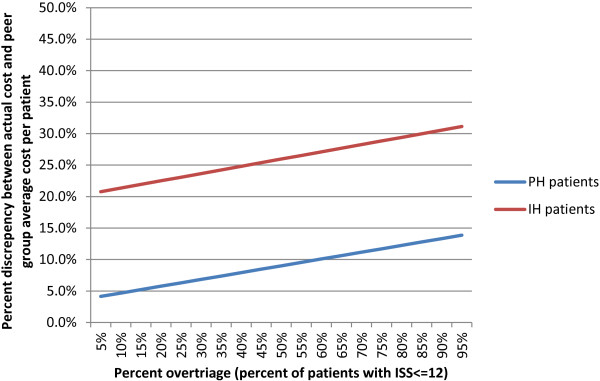
Potential funding discrepancy per patient (difference between actual cost and peer group average cost) for pre-hospital (PH) and inter-hospital (IH) HEMS transfers, weighted by increasing over-triage thresholds (ISS≤12).

Using the estimated funding discrepancy (difference between true cost and peer group average) as a proportion of the actual cost in Table 3, Figure 3 shows a sensitivity analysis of the impact of increasing levels of over-triage (according to local criteria: ISS≤12) for major trauma centres receiving PH and IH patients respectively. For PH and IH HEMS transports, results show a rising discrepancy between the true cost and the peer group average for increasing proportions of over-triage (5%-95% over-triage: 4.1%-13.9% discrepancy [PH]; 20.8%-31.1% discrepancy [IH]).

## Discussion

Helicopter transport is an integral component of modern trauma systems which, in turn, have been shown to reduce preventable mortality in Australia [1,19]. However, few studies have investigated the financial implications of such systems, particularly from the perspective of the hospitals that receive trauma patients. Our study aimed to investigate the financial implications of HEMS over-triage from the perspective of the major trauma centre in NSW. In doing so, we have provided the first state-wide description of HEMS patient characteristics and estimates of HEMS over-triage. In addition to previous literature demonstrating the inadequacy of the episode funding model [20-23], our results highlight the implications of episode funding to a major trauma hospital that receives HEMS patients. Specifically, in terms of potential funding discrepancies, over-triaged HEMS patients may be as costly to a trauma centre as correctly triaged patients. Further, the financial impact of receiving HEMS patients varies by the type of transport undertaken (either pre-hospital or inter-hospital).

In many developed countries, HEMS are used to complement existing ground infrastructure. Recent reviews have shown a consistent association between HEMS use and improved patient outcomes in trauma [24,25]. However, due to the difficulty in accurately determining patient acuity, HEMS patients are frequently over-triaged; resulting in patients being transported who do not require advanced care or expedient transport. Our results demonstrate that HEMS patients in NSW have a high over-triage rate. This is consistent with a meta-analysis of the HEMS literature showed 60% of patients (99% CI: 54.5%-64.9%) transported by HEMS had minor injuries and 25.8% (99% CI: -1.0%-52.6%) were discharged within 24 hours [11].

Patients transported by HEMS in NSW may be over-triaged, however, our data did not allow assessment of transport protocol adherence. In NSW, HEMS are currently activated either by emergency call information (via a rapid launch coordinator) or via on on-scene paramedic according to service protocols, which rely on criteria such as patient physiology and mechanism of injury. In terms of discriminative accuracy, previous research has shown currently used criteria (including injury mechanism, physiology and anatomy of injury) to rely on a limited evidence base [26]. Our results confirm the advanced diagnostic capability and oversight which is possible in inter-hospital transfers lead to patients with a higher acuity being transferred by HEMS. Although such capabilities are not available in the pre-hospital environment and a degree of over-triage is always likely to exist, there remains scope for further research in this area. However, more comprehensive data are needed (linking ambulance to trauma registry and long-term outcomes) to understand which patients benefit from a HEMS intervention and why. From the health system perspective, such data would allow for possible improvements in the cost-effectiveness of a HEMS intervention [12]. Additionally, from the perspective of the receiving hospital, improved HEMS triage would possibly allow more efficient resource allocation to patients who require more of the services offered at a major trauma centre.

During the study period, a HEMS pre-hospital and inter-hospital patient cost, on average, ~$25,000 and ~$42,000 respectively to treat, although considerable variation existed between patients, which has also been demonstrated previously [27]. Our results show HEMS patients are potentially underfunded in the order of ~$2,500 - ~$2,900 per patient transported pre-hospital and inter-hospital respectively. Overall, the potential funding discrepancy was over $1.7 m for the entire year. These results support the need for further research to refine funding models to account for the complexity of trauma patients.

In terms of the cost of over-triage to the major trauma centre, we found treating patients transported by HEMS with minor to moderate injuries (according to the NSW definition; ISS<12) led to a shortfall between the cost of treatment and potential reimbursement of ~$542,000, split evenly between pre-hospital and inter-hospital transports. Previous research has shown more inaccuracy in the episode funding model in less severely injured patients [6] and our results support these findings. Although a proportion of pre-hospital over-triaged patients would have received care at the same centre if transported by other transport modes, HEMS pre-hospital responses in NSW often bypass the closest designated trauma hospital [28]. Therefore, the cost implications of HEMS over-triage to a major trauma centre is a significant consideration.

Another implication of our results is the difference in patient acuity, cost and reimbursement between HEMS patients transported directly from the scene and inter-hospital. Our findings showed patients transported inter-hospital were older, had longer lengths of stayed and consumed more resources, particularly in the ICU. In terms of potential funding discrepancies, our results showed inter-hospital patients with minor injuries had the largest discrepancies compared to patients transported pre-hospital. Currently, hospitals in NSW receive variable amounts of pre-hospital and inter-hospital HEMS transports. Given the differences between HEMS patients transported directly from the scene and inter-hospital, future funding models also need to account for these differences.

This study includes the first comprehensive cohort of patients transported by HEMS to major trauma centres in NSW. However, due to excluded hospitals as well as potentially inconsistent data collection in some included hospitals, our study carries several limitations. We were unable to gather information for hospitals other than major trauma centres (such as regional trauma centres) that receive trauma patients via HEMS. However this is only likely to represent a small proportion of total transports. Some trauma centres did not collect comprehensive data for minor trauma patients, and therefore our data may be an under-representation of this patient group. Finally, it should be noted that our estimate of reimbursement used is based on the peer group averages and do not account for the potential additional weightings for aspects such as public or private status, aboriginality and longer lengths of stay. However, our results are a robust estimate of the true costs of treating HEMS patients relative to the average costs for the same patients among similar peer group hospitals.

## Conclusion

A HEMS brings many advantages to a regionalised trauma system, however their use has implications for receiving hospitals and the broader system. In NSW, HEMS over-triage rates were between 17% and 51% depending on the definition used, which broadly matches results from other jurisdictions. Although further research is required to refine HEMS dispatch criteria, a degree of over-triage is always likely to exist. It suggests that whilst the practice of over-triage is to a large extent driven by a social imperative to insure against the possibility that someone faced with life threatening injuries is under-treated, the trauma centres that provide these services bear much of the burden for this practice. Depending on volume and types of HEMS transports received, this is likely to have variable effects on receiving hospitals in NSW. Future episode funding models therefore need to account for the variability in resource use across different types of trauma patients and the volume of trauma that is transported via HEMS.

## Abbreviations

HEMS: Helicopter Emergency Medical Service; ISS: Injury severity score; PH: Pre-hospital; IH: Inter-hospital; NSW: New South Wales; OR: Operating room; ICU: Intensive care unit.

## Competing interests

The authors declare that they have no competing interests.

## Authors’ contributions

CT and KC conceived this study. CT carried out the statistical analysis and drafted the original manuscript. KC, SJ and MN provided clinical and health service expertise and reviewed the manuscript. All authors read and approved the final manuscript.

## Pre-publication history

The pre-publication history for this paper can be accessed here:

http://www.biomedcentral.com/1471-227X/13/11/prepub
